# Chemical Activation of a Single Melamine Molecule via Isomerization Followed by Metalation with a Copper Atom

**DOI:** 10.1021/acsnano.4c18832

**Published:** 2025-02-26

**Authors:** Karl Rothe, Manex Alkorta, Nicolas Néel, Thomas Frederiksen, Jörg Kröger

**Affiliations:** † Institut für Physik, Technische Universität Ilmenau, D-98693 Ilmenau, Germany; ‡ Centro de Física de Materiales (CSIC-UPV/EHU), E-20018 Donostia−San Sebastián, Spain; ¶ Donostia International Physics Center (DIPC), E-20018 Donostia−San Sebastián, Spain; § Fisika Aplikatua Saila, University of the Basque Country (UPV/EHU), E-20018 Donostia−San Sebastián, Spain; ∥ IKERBASQUE, Basque Foundation for Science, E-48011 Bilbao, Spain

**Keywords:** atomic force microscopy, density functional theory, isomerization, metalation, single molecule

## Abstract

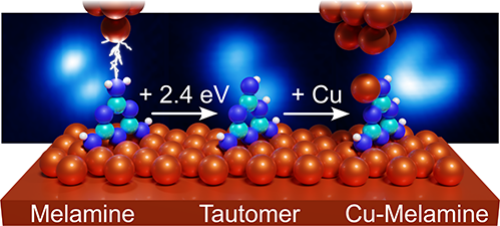

Scanning probe methods have very successfully been used for inducing on-surface reactions and imaging with high resolution the reaction partners at the single-molecule level. However, the entire sequence of chemically activating an educt, identifying its reactive site, running a chemical reaction, and quantifying the involved forces and energies has been missing to date. Here, the organic molecule melamine adsorbed on Cu(100) serves as a single-molecule model system for activation via tautomerization and subsequent metalation with a single Cu atom. An atomic force microscope with a CO-decorated tip probes the most reactive intramolecular site of the tautomer, while a Cu-terminated tip transfers a single Cu atom to this site. Following the interaction between the mutually approached reaction partners up to the verge of chemical-bond formation enables access to the force and energy involved in the single-molecule metalation process. Total-energy calculations from density functional theory support the experimental findings and illustrate the structure of the reactants.

The atom-by-atom manipulation of matter belongs to the perhaps most fascinating capabilities of a scanning tunneling microscope (STM) and an atomic force microscope (AFM). After the first reports of atomic manipulation^1^ and controlled assembly of atoms,^2^ the fabrication of artificial atomic and molecular structures has seen a tremendous development, which is well described in review articles.^3−11^ Today, chemical reactions can be induced at the single-molecule level,^12,13^ new quantum states engineered,^14^ and artificial-atom or artificial-molecule orbitals simulated.^15^ The termination of the scanning probe with single atoms or molecules has certainly contributed to the contemporary understanding of quantum chemistry at surfaces. By attachment of an O atom to the W tip apex, chemical specificity was added to STM already in early experiments.^16^ Moreover, the spatial resolution of STM strongly benefited from decorating the tip with a variety of molecules,^17−33^ in particular in the Pauli repulsion distance range between the tip and surface of STM^34−36^ and AFM^37−46^ junctions.

The intentional decoration of the STM or AFM probe surmounts the sheer high-resolution imaging capability by far. For instance, in vibrational spectroscopy with STM, the termination of the tip defines the symmetry of the most transmitting electron transport channel and, thus, the excitation and detection of quantum vibrations.^47−56^ In addition, spin excitations can be explored at the single-atom limit by appropriately functionalized probes.^57−60^ In quantum-chemistry experiments, the AFM tip has become an indispensably active part in probing the interaction with a reaction partner,^61^ physisorbed and chemisorbed states,^62,63^ relaxations in molecular contacts,^64,65^ and chemically reactive sites^66−69^ and in controlling nonequilibrium bond forces.^70^

The combined STM/AFM and density functional theory (DFT) studies presented here were in part stimulated by the central role of melamine (2,4,6-triamino-1,3,5-triazine, C_3_H_6_N_6_, MH; Figure 1) in the fabrication of supramolecular networks,^71−76^ in particular when the binding to metal atoms is involved.^77−79^ Moreover, the complete sequence comprising the chemical activation of a reactant, the identification of its reactive site with intramolecular resolution, the formation of a chemical bond, and the measurement of force and energy involved in the reaction has so far not been reported in a single experiment. The findings of the work demonstrate that adsorbed MH on Cu(100) represents an ideal model system to scrutinize the metalation process at a fundamental level by surveying the reaction between the two bonding partners, a single MH tautomer and a single Cu atom.

**Figure 1:**
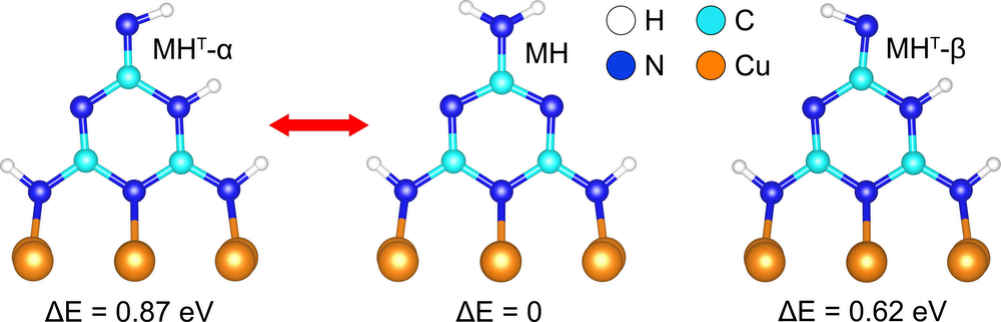
Relaxed structures of melamine (MH, middle) and its tautomers (MH^T^-α, left, and MH^T^-β, right) on Cu(100). The calculated total-energy differences Δ*E* relative to MH are indicated. On-surface tautomerization induces the reversible hopping of a H atom from the top amino group to an N atom of the 1,3,5-triazine ring. The two tautomer variants differ in the position of the remaining top H atom. The arrow indicates the reversible tautomerization assigned to the experimental observations.

## Results/Discussion

**Figure 2:**
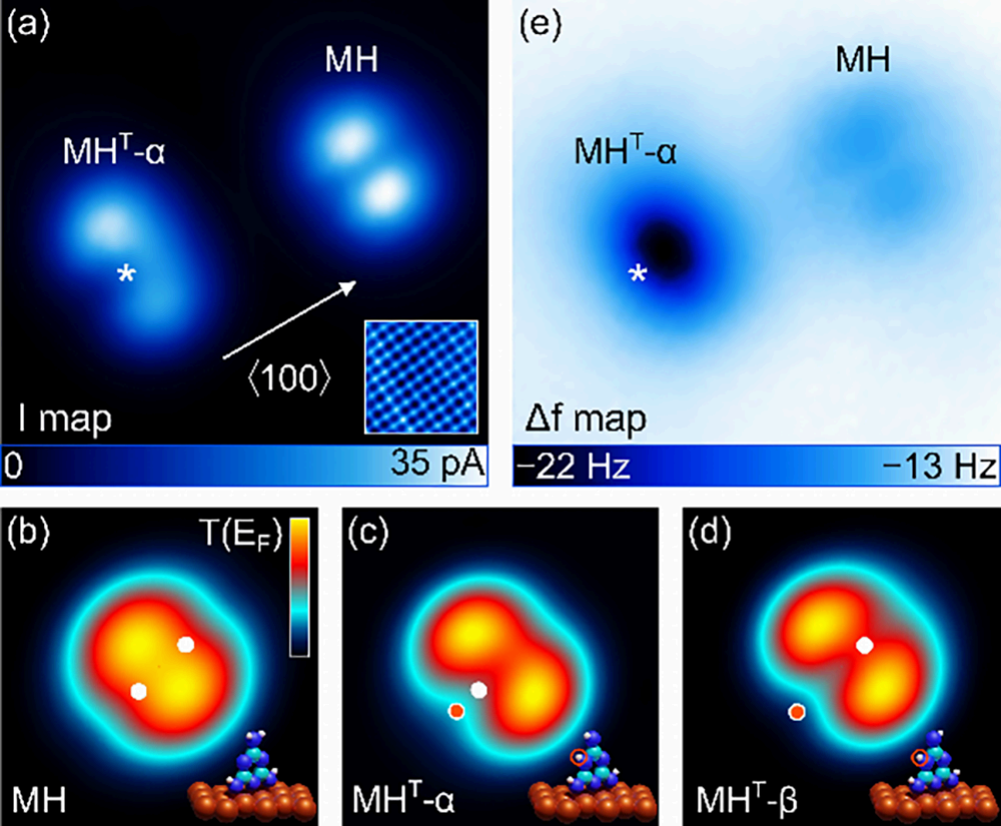
(a) Metal-tip constant-height *I* map of indicated MH and MH^T^-α molecules adsorbed on Cu(100) (2.1 nm × 2.1 nm). Inset: Atomically resolved STM image of the Cu(100) lattice (100 mV, 250 pA, 2 nm × 2 nm). (b–d) Calculated maps of *T*(*E*_F_) for MH, MH^T^-α, and MH^T^-β (1 nm × 1 nm) at tip excursion in the tunneling range (Δ*z* = −300 pm; see the text). Dots mark the position of H atoms (white, unaffected H atoms; red, displaced H atoms due to tautmerization). Insets: Pseudo-three-dimensional views of the relaxed adsorption geometries. (e) Map of Δ*f* simultaneously recorded with part a. In parts a and e, the H atom that remained at the top N atom after tautomerization is marked with an asterisk. For the simultaneously recorded maps, the feedback loop had been deactivated at 100 mV and 50 pA above pristine Cu(100). The sample voltage was then reduced to 10 mV and the tip retracted by 50 pm.

Room-temperature deposition of MH on Cu(100) gives rise to the upright adsorption of the molecule^80^ where two amino groups lose one H atom each, thereby enabling a chemical bond of three N atoms to the substrate (Figure 1, middle). In constant-height current (*I*) maps recorded at low sample voltage (*V*), MH (right side of Figure 2a) appears with *C*_2*v*_ symmetry showing two protrusions symmetrically positioned on two sides of a linear depression. The calculated electron transmission [*T*(*E*_F_)] map for MH evaluated at the Fermi energy (*E*_F_; Figure 2b) reproduces the *C*_2*v*_ symmetry and the central nodal line of the STM data. The central linear depression can therefore be assigned to the MH backbone that is oriented along a crystallographic ⟨100⟩ direction of Cu(100), which was inferred from the atomically resolved substrate lattice (inset to Figure 2a). Furthermore, the protrusions to both sides of the molecular plane are associated with electron transport through the π orbitals of MH. While the comparison of simulated transmission maps with STM images must be treated with care,^81,82^ the presented results are likewise in line with previous conclusions on the basis of simulated constant-current STM images.^80^

The on-surface tautomerization of MH is induced by the local injection of electrons with elevated energy (2.4 eV) from the AFM metal tip at the center of the MH backbone.^80,83^ The *I* map of the tautomer (left side of Figure 2a) shows the reduction of the former *C*_2*v*_ to a single mirror symmetry. Indeed, the central linear depression is no longer uniform as in the case of MH. Rather, it has a bright and dim end giving rise to an overall crescent shape of the imaged molecule. Total-energy calculations from DFT offer two tautomer structures, MH^T^-α and MH^T^-β (Figure 1), which are close in energy. The associated calculated *T*(*E*_F_) plots of MH^T^-α (Figure 2c) and MH^T^-β (Figure 2d) both reproduce the single mirror symmetry of the experimental data. However, the crescent-shaped appearance in the *I* map of the tautomer is more closely reproduced by the simulated MH^T^-α *T*(*E*_F_) plot, which is due to both the top and tautomerized H atom being located on the same side of the molecule. For MH^T^-β, these two H atoms reside at opposite sides of the tautomer, which results in an almost uniform nodal line in the *T*(*E*_F_) map. These observations are in accordance with previous assignments due to simulations of STM images^80^ and allow a first tentative assignment of the imaged tautomer to MH^T^-α. This assignment will further be corroborated below by high-resolution imaging with a CO-terminated tip and by metalation of the tautomer. It is noteworthy that in the noncontact frequency modulation AFM image (Figure 2e) simultaneously recorded with the *I* map (Figure 2a), an extended depression occurs on the side of MH^T^-α that appears as a protrusion in the *I* map; that is, an increased attraction is probed on the side of the tautomer exhibiting the dangling bond of the top N atom. Below, this intramolecular site will be analyzed in a quantitative manner and used for metalation. The intact MH, in contrast, appears with very faint contrast in the AFM topograph.

**Figure 3:**
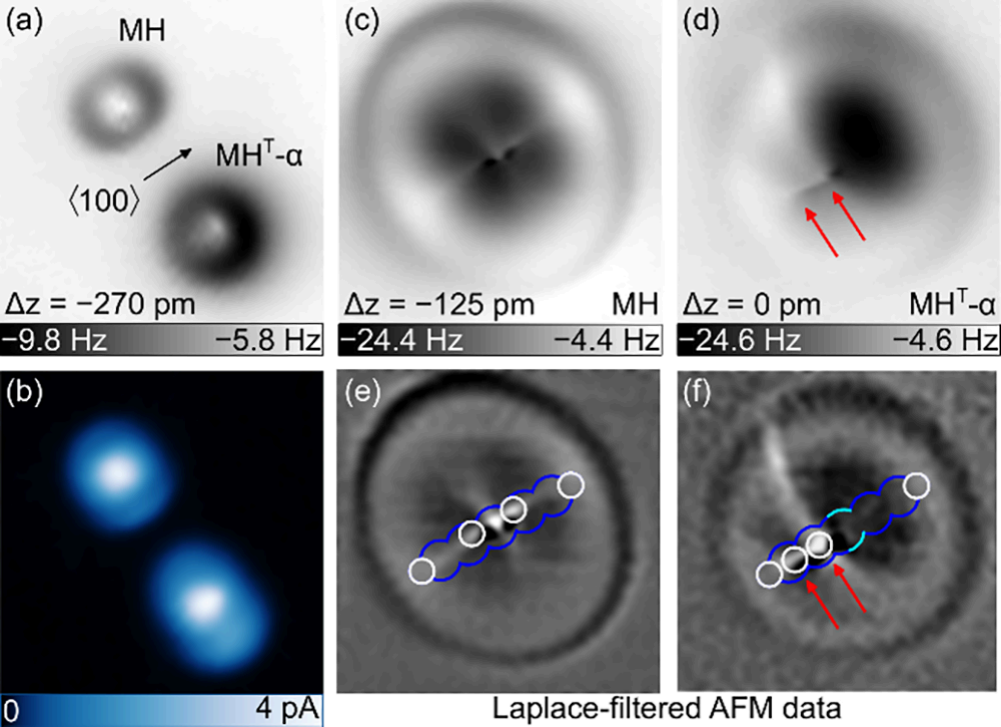
(a) Constant-height map of resonance frequency change Δ*f* measured with a CO-terminated AFM probe above MH and MH^T^-α at large tip–molecule separation (10 mV, 2.1 nm × 2.1 nm). The arrow marks a ⟨100⟩ direction, which applies to all topographs. (b) Constant-height *I* map simultaneously recorded with part a. (c) Close-up view of MH in part a at a small tip–molecule distance (10 mV, 1.2 nm × 1.2 nm). (d) As part b for MH^T^-α. (e) Laplace-filtered AFM data of part c with superimposed atom contours of the simulated relaxed MH (white, H atoms; blue, N atoms). (f) Laplace-filtered AFM data of part d with superimposed atom contours of the simulated relaxed MH^T^-α (light blue: C atom). Arrows in parts d and f mark the positions of the topmost H atoms.

To additionally confirm the assignment of the experimentally produced tautomer to MH^T^-α, AFM images were acquired with a CO-decorated tip. Termination of the AFM tip with a single CO molecule was achieved by a standard routine.^84^Figure 3a shows that MH in constant-height maps of the probe resonance frequency change (Δ*f*) at large tip–molecule distance gives rise to a central protrusion that is surrounded by a dark rim while the central protrusion for the tautomer is less pronounced and asymmetric as well as encircled by a wider dark ring than observed from MH. In the simultaneously recorded *I* map (Figure 3b), both MH and MH^T^-α exhibit a bright center and less bright protrusions at mirror-symmetric sides of the center. Close-up views of Δ*f* maps at even lower tip–molecule distance show submolecular details, as expected for a CO tip in the Pauli repulsion distance range.^37,85,86^ The molecular backbone of MH (Figure 3c) appears as a faint bright line parallel to ⟨100⟩, while the frontier π orbitals give rise to a similarly weak protruding feature oriented perpendicular to the MH plane. These two lines give rise to a cloverlike pattern of depressions reflecting the *C*_2*v*_ symmetry of the MH-Cu(100) adsorption complex. In contrast, the AFM image of the tautomer (Figure 3d) exhibits a single mirror symmetry only and is dominated by a pronounced depression that signals an intramolecular attractive region, in agreement with AFM images acquired with a Cu tip at large tip–molecule separation (Figure 2e). On the opposite side of the depression, protrusions occur at the positions of the MH^T^-α backbone that are compatible with the sites of the topmost H atoms (arrows). To see this site assignment more clearly, Laplace-filtered data of MH (Figure 3e) and MH^T^-α (Figure 3f) are superimposed with the top-view atomic contours of the respective relaxed molecule structures (Figure 1). The calculated positions of the topmost H atoms of MH agree well with the regions of brightest contrast in the Laplace-filtered AFM data. Importantly, also for the tautomer, the topmost H atoms of the simulated MH^T^-α coincide with the AFM protrusions (arrows in Figure 3d,f). Rather than the H atoms themselves, the N–H bonds are more likely to appear in the AFM maps due to their increased charge density.^37,44,87−89^

**Figure 4:**
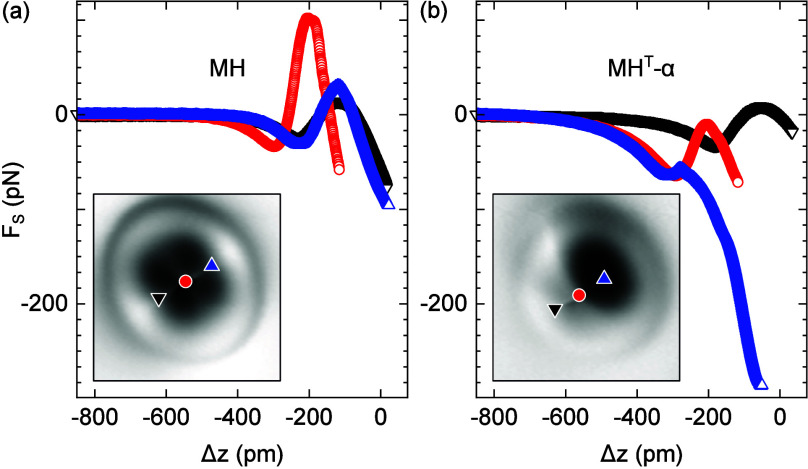
(a) Spatially resolved variation of short-range force *F*_s_ with CO tip excursion Δ*z* atop the indicated sites (symbols in the inset) of MH [Δ*z* = 0 is defined by feedback loop parameters 100 mV and 50 pA above Cu(100)]. (b) As part a for MH^T^-α.

To quantitatively explore the attraction of MH and MH^T^-α to possible reaction partners, the same CO-functionalized tip was approached with picometer resolution toward different intramolecular sites and the short-range force (*F*_s_) between the oscillating CO probe and the molecule was extracted from simultaneously recorded Δ*f* versus Δ*z* traces (Figure S1).^70,90^ Comparing the experimental *F*_s_ variations for MH (Figure 4a) and MH^T^-α (Figure 4b), it is obvious that, for all tip excursions, MH and MH^T^-α exhibit nearly the same attraction to CO, unless *F*_s_ is probed above the top H-abstracted N atom of MH^T^-α (upright triangle in the inset to Figure 4b). Above this site, at the maximum tip excursion Δ*z* = −52 pm, the short-range attraction of MH^T^-α to the CO probe is more than a factor 10 larger than observed from MH at the same Δ*z*, i.e., −287 pN (MH^T^-α) compared to −27 pN (MH). This difference is indicative of an increased chemical activity of MH^T^-α at the tautomerization site, which is corroborated by the associated dark region in AFM maps (Figure 3c) and by the controlled metalation at this site (*vide infra*). Spatially resolved *F*_s_ data with an increased number of intramolecular sites are presented in Figure S2.

The *F*_s_ versus Δ*z* evolution for both MH and MH^T^-α does not follow the variation expected from a simple Lennard-Jones interaction potential between CO and the adsorbed molecule. Rather, in case of MH an attractive *F*_s_ minimum precedes a repulsive region of *F*_s_, which then turns into a steep decrease. For MH^T^-α a similar variation of *F*_s_ is observed. However, the repulsive maximum is very weak (Figure 4b, *F*_s_ traces acquired at the lower left boundary and at the center). The *F*_s_ evolution at the tautomerization site, however, continues a steep attraction after traversing a shallow minimum without subsequent repulsion. This peculiar behavior can be rationalized in terms of a bending of the functionalized probe^91,92^ (Figure S3), possibly in combination with vertically successive physisorption and chemisorption states^62,66^ and relaxations of the adsorbed molecule.^63,69^

**Figure 5:**
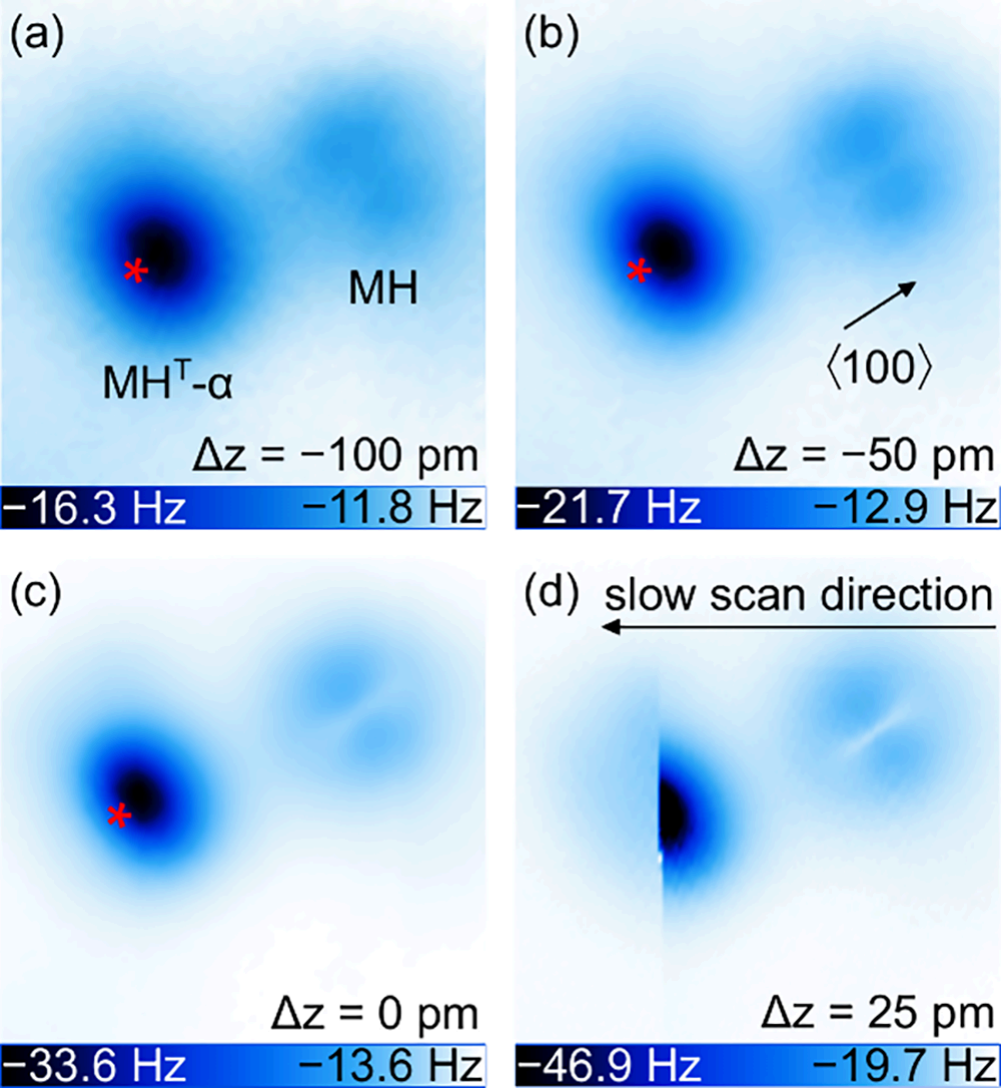
Gallery of constant-height Δ*f* maps acquired with a Cu tip atop MH and MH^T^-α with decreasing tip–surface distance from part a to part d (2.1 nm × 2.1 nm). Zero tip excursion is defined by the tip–surface separation at the feedback loop parameters 100 mV and 50 pA above Cu(100). The asterisks in parts a–c mark the position of the top H atom. The image distortion in part d for MH^T^-α is due to Cu atom transfer from the tip to the molecule (see text).

The experimental verification of the increased chemical reactivity of the tautomerized MH^T^-α site proceeded via a Cu atom, a prominent bonding partner for melamine in its complexes with metals.^75,93−96^ To this end, the CO molecule had been removed from the AFM probe,^84^ which was subsequently terminated with a single Cu atom by applying previously reported procedures.^97−99^ The general appearance of MH with *C*_2*v*_ symmetry and faint contrast as well as MH^T^-α as a dark depression (Figure 2e) does not change in a notable manner upon approaching the Cu tip (Figure 5a–c). Importantly, at Δ*z* = 25 pm imaging of MH^T^-α becomes unstable (Figure 5d), which is reflected by a sudden change in the Δ*f* signal. Such image distortions are independent of the scan direction. They reproducibly occur when the Cu tip comes close to the intramolecular attractive region of MH^T^-α. In contrast, the image of MH remains undistorted.

**Figure 6:**
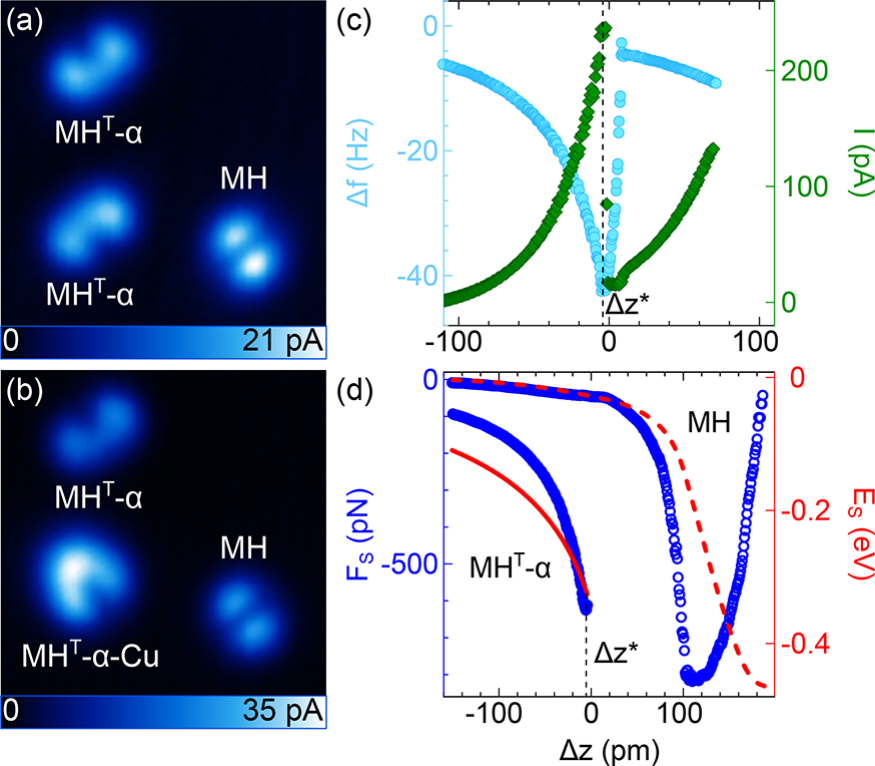
Single-melamine metalation and quantitative measure of force and energy involved. Constant-height *I* maps of MH and MH^T^-α recorded with a Cu tip (a) before and (b) after metalation (2.7 nm × 2.7 nm). The feedback loop had been deactivated above Cu(100) at 100 mV and 50 pA before tip retraction by 75 pm for mapping *I* at 10 mV. (c) Resonance frequency change Δ*f* as a function of tip excursion Δ*z* (dots) showing the abrupt variation at the tip–MH^T^-α contact, where likewise *I* (lozenges) deviates from its exponential increase. (d) Short-range force *F*_s_ and associated energy *E*_s_ as a function of tip displacement Δ*z* toward MH^T^-α (dots, solid line) and MH (circles, dashed line). Tip excursion Δz* (dashed lines in parts c and d) marks the discontinuous change in Δ*f* and *I*. Feedback loop parameters 100 mV and 50 pA define Δ*z* = 0 in parts c and d.

The origin of the behavior experienced in imaging MH^T^-α at small tip–molecule distances is unveiled in local Δ*f* spectroscopy experiments where the Cu tip approaches MH and MH^T^-α central regions and the Δ*f* signal is simultaneously recorded. Figure 6 depicts constant-height *I* maps of one MH and two MH^T^-α molecules prior to (Figure 6a) and after (Figure 6b) approaching the Cu tip toward the bottom MH^T^-α to an extent similar to the tip excursions where distorted images (Figure 5d) occur.

In the associated Δ*f* trace recorded atop MH^T^-α (Figure 6c), Δ*f* changes abruptly at Δz* signaling contact formation.^68,100^ It coincides with an order-of-magnitude drop of the current. Retracting the tip after these abrupt changes of Δ*f* and *I*, the appearance of MH^T^-α in *I* maps has changed substantially (Figure 6b). Its maximum apparent height increased by a factor 3 at the intramolecular region that coincides with the depression in Δ*f* maps (Figure 3c). This observation is assigned to the Cu metalation of MH^T^-α, i.e., to the transfer of a single Cu atom from the tip to the molecule, which is referred to as MH^T^-α-Cu in the following. Such single-atom transfers are well-known from surfaces,^97^ demetalation^101^ and metalation of phthalocyanines.^68,102^ They rely on the coating of the tip apex with substrate material, which is ensured by the repeated indentation of the tip into the clean substrate surface in the course of tip preparation, and on the atomic-scale relaxations of the junction geometry due to strong adhesive forces at small tip–surface distances.^97,103^ The entailed atomic fracture of the tip is quantified in Figure S4. This process requires a mechanical force, from which a lower bound to the metalation force can be extracted. To this end, the evolution of the short-range force *F*_s_ with Δ*z* is presented for MH^T^-α (left part of Figure 6d). It gives rise to *F*_s_* ≡ *F*_s_(Δz*) = −569 ± 106 pN, where the values represent arithmetic means and standard deviations from four metalation reactions with different tips (Figure S5). The rather small standard deviation reflects the reproducibility of the metalation, which is further corroborated by the sequential metalation of two adjacent MH^T^-α molecules (Figure S6). Figure 6d likewise shows the associated short-range energy *E*_s_, which results from the numerical integration of *F*_s_. This approach to *E*_s_ is valid if the underlying short-range force *F*_s_ is conservative. The latter is applicable because the phase of π/2 between the driving force and deflection of the oscillating probe ensures the decoupling of conservative and dissipative forces.^104,105^ Moreover, in this case, the Δ*f* versus Δ*z* trace contains the information on the conservative force only.^106^ In addition, the dissipation signal is essentially constant throughout the entire tip approach to MH and up to the point just before the incipient bond to MH^T^-α. This observation is rationalized in terms of elastic junction relaxations that are reversible within this range of tip excursions, that is, no energy is dissipated due to irreversible deformations of the contact. As a result, *E*_s_* ≡ *E*_s_(Δz*) = −0.29 ± 0.04 eV. A similar approach was previously applied to quantify the force and energy involved in the tautomerization of a porphycene^107^ and the metalation of a phthalocyanine.^68^

Before presenting the accompanying calculations that corroborate the suggested reactivity of MH^T^-α, the evolution of *F*_s_ observed from MH (right part of Figure 6d) is noteworthy. It contrasts the behavior of MH^T^-α in that the point of maximum attraction is entirely traversed without discontinuity. Tip retraction from these and even closer tip–MH separations leaves the structural integrity of probe and molecule invariant, as seen from subsequent microscopic images. Consequently, adsorbed MH is inert to Cu metalation, in strong contrast to its tautomerized counterpart.

**Figure 7:**
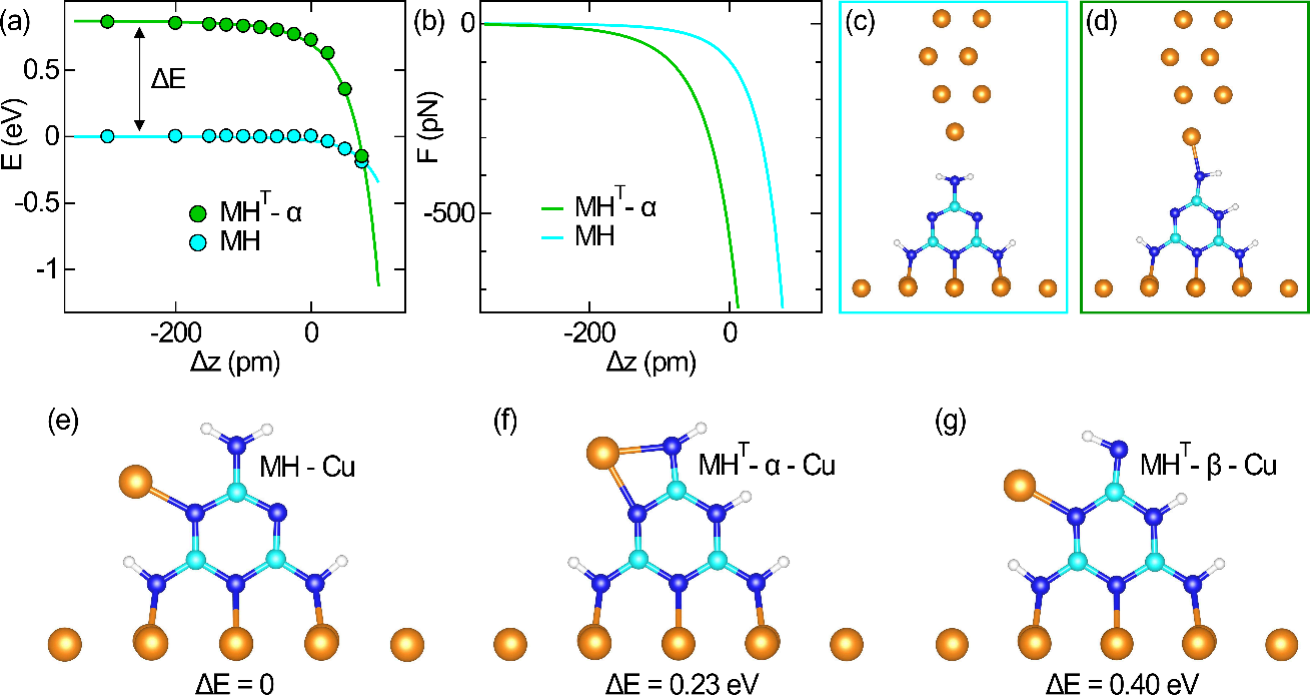
Simulation of single-melamine junctions. (a) Total energy *E* calculated for a single-atom-terminated Cu model tip approaching a single MH (bottom) and MH^T^-α (top) on Cu(100) by Δ*z* (Δ*z* = 0 is defined as Δz*; Figure 3b). (b) Force *F* resulting from a Lennard-Jones potential fit to the total-energy variation in part a. (c) Relaxed single-MH junction at Δ*z* = 75 pm. (d) As part c for MH^T^-α. The Cu–N bond has already formed. (e–g) Different metalation products with the indicated total-energy difference relative to MH-Cu.

DFT calculations were performed to elucidate the tip–isomer interaction and to unveil at the atomistic level potential geometries of the metalation product. To this end, the total energy *E* of a junction comprising an idealized Cu metal tip and the relaxed MH and MH^T^-α on a Cu(100) surface was computed (Figure 7a). In the far tunneling range, the two isomer adsorption complexes are separated by Δ*E* = 0.87 eV (Figure 1). While tip approach to MH up to Δ*z* ≈ 0 leaves *E* of the MH junction nearly constant, an identifiable lowering of *E* starts to occur already at Δ*z* ≈ −100 pm for the MH^T^-α junction. The subsequent decrease in energy is more pronounced for MH^T^-α than for MH, the former even diving below the MH junction energy at Δ*z* ≈ 75 pm. The associated attractive force (Figure 7b) was derived from the numerical derivative of the Lennard-Jones potential function that was fit to the calculated *E* profile (solid lines in Figure 7a). Clearly, the tip–MH^T^-α attraction is stronger than the attraction between tip and MH for Δ*z* > −200 pm. In particular, at contact (Δ*z* = 0) the attraction is nearly 6 times larger for MH^T^-α than for MH. This result is in very good agreement with the experimental findings (Figure 6d). Parts c and d of Figure 7 show MH and MH^T^-α junctions with the same small tip–molecule distance. While a chemical bond between the Cu apex atom of the tip and MH, i.e., its metalation, is impeded, the bond has already formed for MH^T^-α at the unsaturated dangling bond of the top N atom. These simulations support the experimental observations and, thereby, further corroborate the assignment of MH^T^-α to the actual tautomer.

Going beyond the Cu–N bond formation in MH^T^-α-Cu, the simulations additionally addressed the important question of the configuration of the final metalation product. To this end, the energy landscape for different isomer-Cu compounds was explored. As reasonable reactants, MH-Cu (Figure 7e), MH^T^-α-Cu (Figure 7f), and MH^T^-β-Cu (Figure 7g) were considered among others (Figure S7). The Cu atom was incorporated into all considered MH isomers and the topmost H atoms were interchanged for spotting possible transition states in the metalation. The simulations show that MH-Cu (Figure 7e) is energetically preferred to MH^T^-α-Cu by 0.23 eV and to MH^T^-β-Cu by 0.40 eV.

Although MH^T^-α-Cu is energetically less favored than MH-Cu in the simulations, preference is given to the former tautomer-Cu compound for the following reasons. Constant-height *I* maps of the reaction product (Figure 6b) show a horseshoe motif with the open side appearing dark. It is reasonable to assign this region to the side of MH^T^-α where the top H atom, the tautomerized and the bottom H are lined up. In addition, once the Cu atom binds to the top N atom (Figure 7d), it is very close to the N atom of the triazine ring to which a chemical bond can readily be formed, which is consistent with the extended intramolecular region of attraction (Figure 3c).

For all simulated final configurations of the metalation reaction, calculated transmission maps show a strong signal at the Cu site (Figure S8), which results from the ample overlap of Cu *s*-orbitals at the tip and the tautomer. Therefore, the brightest part of MH^T^-α-Cu in *I* maps (Figure 6b) can be associated with the transferred Cu atom.

Before concluding, the applicability of the methods presented in this work to other molecules and surfaces is briefly discussed. A favorable property of the model system used in our studies is the upright adsorption of the molecule. It allows the chemical activation of a site that is exposed to vacuum and whose entailed dangling unsaturated bonds do not hybridize with the surface, irrespective of the nature of the surface. For molecules adopting a flat adsorption geometry, i.e., a configuration where all molecular moieties are close to the surface, the dangling bonds arising from the activation may quickly be saturated by hybridization with the surface atoms. In this case, not all surfaces may prove suitable, and inert dielectric materials may be the preferential choice for the single-molecule investigations reported here.

## Conclusions

The combination of STM, AFM, and DFT was successfully deployed to gain a thorough picture of a metal–organic reaction at the single-atom and single-molecule scale. To this end, melamine on Cu(100) was identified as an appropriate model system that allowed to execute all consecutive steps from the initial reactant to the final product. The chemical activation was induced by tautomerization, which was followed by probing the intramolecular reactive site and its subsequent metalation. These individual steps relied on the intentional functionalization of the tip for high-resolution imaging as well as for the active supply of the atomic reactant for metalation. The mechanical fracture of the tip in the course of the metalation provided a quantitative measure for a lower bound to the force and energy involved.

## Methods/Experimental

### Experimental Details

A combined STM and AFM setup was operated in ultrahigh vacuum (5 × 10^–9^ Pa) and at low temperature (4.8 K). Surfaces of Cu(100) were cleaned by Ar^+^ (purity of Ar gas 99.999%) ion bombardment and annealing. Tungsten wire (purity 99.99%, diameter 50 μm), chemically edged in a 0.1 M NaOH electrolyte, was used as the tip material and presumably coated with substrate material (Cu) in the course of in situ tip preparation, which includes tip–surface contacts. The clean surface was exposed at room temperature to MH sublimated from a powder (purity 98%) in a heated (320 K) Ta crucible. Subsequently, CO molecules for tip termination were adsorbed on the MH-covered surface at low temperature (6 K) by backfilling the vacuum vessel with gaseous CO (purity 99.97%) at a partial pressure of 10^–7^ Pa. The transfer of a single CO molecule from the surface to the tip followed a standard routine,^84^ i.e., by approaching the adsorbed CO with the clean tip at elevated sample voltage (4 V). Detachment of CO from the tip was achieved by applying −4 V and approaching the tip to clean Cu(100). For AFM maps acquired in the constant-height noncontact frequency modulation mode, the qPlus sensor^108,109^ was driven at a resonance frequency of 30.5 kHz with a quality factor of 40000 and an amplitude of 50 pm. The vertical force between the tip and sample was extracted from distance-dependent resonance frequency changes.^110,111^ STM data were recorded in constant-current and constant-height mode with the voltage applied to the sample. Data obtained in AFM and STM experiments were processed using WSxM.^112^

### Computational Details

Our DFT calculations are performed with a 3 × 3 Cu(100) surface cell using TranSiesta(113−115) within the generalized gradient approximation.^116^ A double-ζ plus polarization atomic basis was used for the adsorbate structure (C, H, N, and Cu) as well as for the Cu apex atom of the tip, and a single-ζ plus polarization atomic basis for the remaining Cu atoms. Pseudopotentials and basis radii were taken from the SIMUNE Atomistics data set.^117^ A 400 Ry energy cutoff was used for the real-space grid integrations. Relaxations were performed until residual forces were smaller than 0.01 eV/Å. Using a harmonic vibrational analysis, we checked that the three melamine–metal complexes in Figure 7e–g are metastable. In the transport setup, self-energies take into account the semi-infinite extension of the electrodes in the transport direction, while the transverse (surface) directions are sampled on a 3 × 3 *k* grid while elastic transmissions were computed with Tbtrans on a finer 10 × 10 *k* mesh.

## Supporting Information Available

Additional data on short-range forces, spatially resolved short-range forces, bending of the CO-terminated probe, tip changes upon metalation, simulated tautomer and tautomer-Cu compounds, and calculated constant-height transmission maps (PDF)
