# Supplementary material for: Chemical Activation of a Single Melamine Molecule via Isomerization Followed by Metalation with a Copper Atom
Source: ACS Nano. 2025 Feb 26;19(9):9207–15. doi: 10.1021/acsnano.4c18832 (PMC11912577; doi:10.1021/acsnano.4c18832)
Supplement: Supplementary file 1 — nn4c18832_si_001.pdf [file nn4c18832_si_001.pdf]

Supporting Information
Chemical activation of a single melamine molecule via isomerization followed by metalation with a copper atom

Karl Rothe,[†] Manex Alkorta,[‡,§] Nicolas Néel,[†] Thomas Frederiksen,[¶,‖] and Jörg Kröger[*,†]

†*Institut für Physik, Technische Universität Ilmenau, D-98693 Ilmenau, Germany*

‡*Centro de Física de Materiales (CSIC-UPV/EHU), E-20018 Donostia – San Sebastián, Spain*

¶*Donostia International Physics Center (DIPC), E-20018 Donostia – San Sebastián, Spain*

§*Fisika Aplikatua Saila, University of the Basque Country (UPV/EHU), E-20018 Donostia - San Sebastián, Spain*

‖*IKERBASQUE, Basque Foundation for Science, E-48011 Bilbao, Spain*

E-mail: joerg.kroeger@tu-ilmenau.de

Short-range forces

In order to access short-range forces that are involved in the metalation, long-range and slowly varying attractive forces between the mesoscopic tip structure and the surface must be removed from the experimentally obtained total vertical force. The following approximation has to be made to this end. The measured total vertical force acting on the tip, F, can be decomposed into a short-ranged force between the atom at the tip apex – O in case of a CO-terminated tip (Figure 4) or Cu in case of a Cu-terminated tip (Figure 6d) – and the melamine molecule adsorbed on Cu(100), F_s, and a residual long-ranged, slowly varying force, F_0, acting between the entire tip and the metal substrate. One may expect F_s to

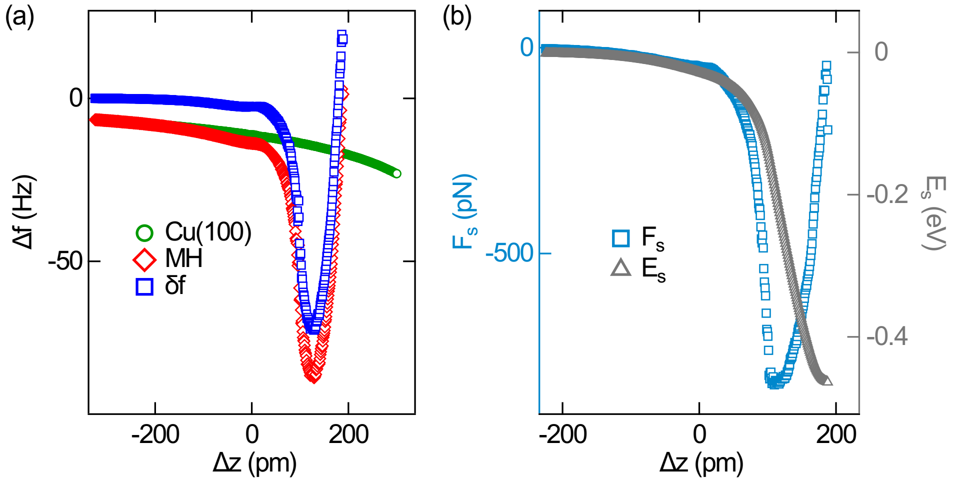

Figure S1: (a) Resonance frequency changes Δf of the force sensor based on a Cu tip measured atop clean Cu(100) (circles) and adsorbed MH (lozenges). The difference of these data sets is denoted δf (squares). Feedback loop parameters 100 mV and 50 pA above Cu(100) define $\Delta z = 0$. (b) Short-range force (F_s, squares) and energy (E_s, triangles) extracted from δf in (a), see text.

be negligible at large distance and dominant at chemical-bond distance. To obtain F_s and test this approximation, a previously suggested and successful procedure[1–3] was applied. The resonance frequency changes were acquired with a Cu-terminated tip on an extended pristine Cu(100) surface region (circles in Figure S1a) at the same absolute tip–surface distance as used for the $\Delta f(z)$ measurements atop adsorbed MH on Cu(100) (lozenges).

These frequency changes are referred to as Δf_0 and reflect the long-ranged slowly varying force F_0. Transforming then $\delta f = \Delta f - \Delta f_0$ (squares) into a force[4,5] results in F_s (squares in Figure S1b). Integration of F_s, *i. e.*, $\int F_s(\Delta z)\,\mathrm{d}\Delta z$, yields the energy E_s (triangles).

Spatially resolved short-range forces

The increased attraction of the tautomer MH^T-α intramolecular site was further corroborated by exploring the spatial evolution of F_s-versus-Δz traces recorded with a CO-terminated tip. Figure S2 compares data for MH (Figure S2a) and MH^T-α (Figure S2b) for a direction

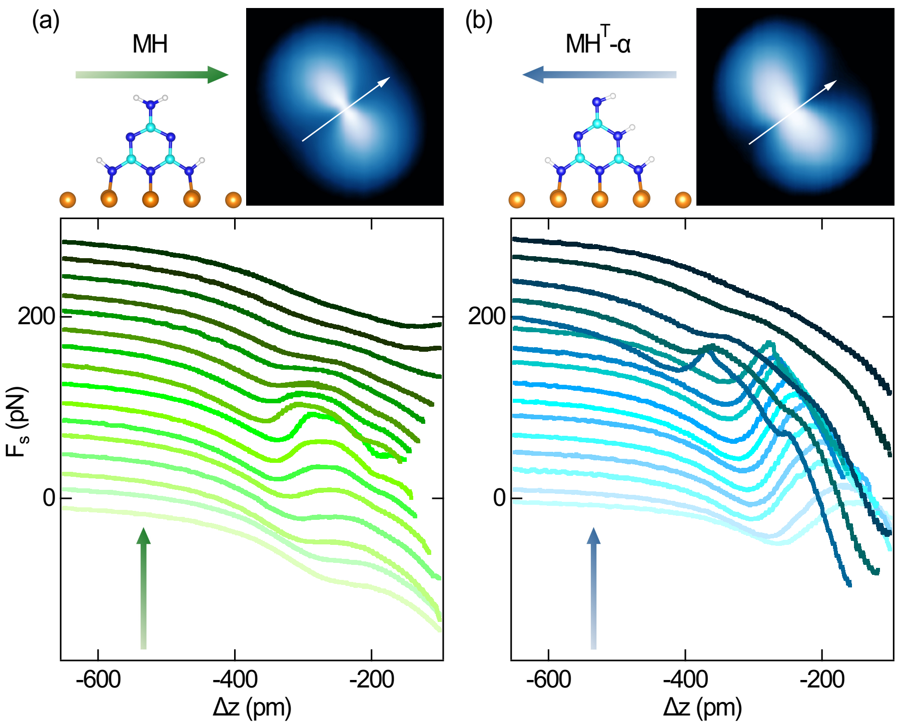

Figure S2: (a) Evolution of short-range force F_s acquired with a CO tip in a direction parallel to the molecular plane of MH, which coincides with ⟨100⟩. Force data excluding the bottom trace are vertically offset by 20 pN. Left inset: side view of the relaxed adsorption structure of MH with arrow marking the order of F_s traces from bottom to top. Right inset: constant-height I map of MH (10 mV, 1 nm × 1 nm) with arrow indicating the sites and sequence of data acquisition. The color scale ranges from 0 (dark) to 67 pA (bright). (b) As (a) for MH^T-α with the color scale of the I map ranging from 0 (dark) to 98 pA (bright).

parallel to the molecular backbone including the intact (MH) and H-abstracted (MH^T-α)

amino group. For MH, the evolution of F_s traces upon approaching the amino group is very similar to the evolution upon departing from it again, which can be seen by the shift of the dip-hump pair first to lower Δz up to the amino group and then back to higher Δz away from the amino group. This variation reflects the mirror symmetry of the MH-Cu(100) adsorption complex. The situation for MH$^\mathrm{T}$-α is markedly different. Approaching the H-abstracted amino group of the tautomer and going beyond is accompanied by a shift of the dip-hump pair to lower Δz, which is particularly strong atop the tautomerized site. Only after passing the site of the missing H atom the attenuated dip-hump pair shifts back to larger Δz.

Bending of the CO-terminated probe

Extending a previously reported simulation of the CO bending at an AFM tip,[6] the peculiar force variations with the probe–molecule distance can qualitatively be reproduced. To this end, the interaction of the CO tip and the MH molecule is reduced to the interaction between the O atom of the probe (at $\mathbf{r}_0$) and three N atoms (at $\mathbf{r}_1$, $\mathbf{r}_2$, $\mathbf{r}_3$) of MH residing on the side of the triangular backbone facing the CO probe (Figure S3a). The interaction potential therefore comprises the Lennard-Jones potential

$$V_{\mathrm{LJ},i} = \varepsilon \cdot \left[\left(\frac{r_\mathrm{eq}}{r_i} \right)^{12} - 2 \left(\frac{r_\mathrm{eq}}{r_i} \right)^{6} \right] \quad (i = 1, 2, 3) \tag{S1}$$

between the O atom of the probe and the three N atoms with $r_i = |\mathbf{r}_i - \mathbf{r}_0|$ and r_eq the equilibrium bond distance. In addition, the harmonic potential

$$V_\mathrm{rot} = \frac{1}{2} k \ell^2 \sin^2 \varphi \tag{S2}$$

describing the CO bending at the tip[7] with k the lateral spring constant, ℓ the CO equilibrium bond length, φ the angle between the surface normal and the C–O axis (Figure S3a) enters

the simulations. Long-range van der Waals and electrostatic interactions were not considered because of their removal from experimental data.

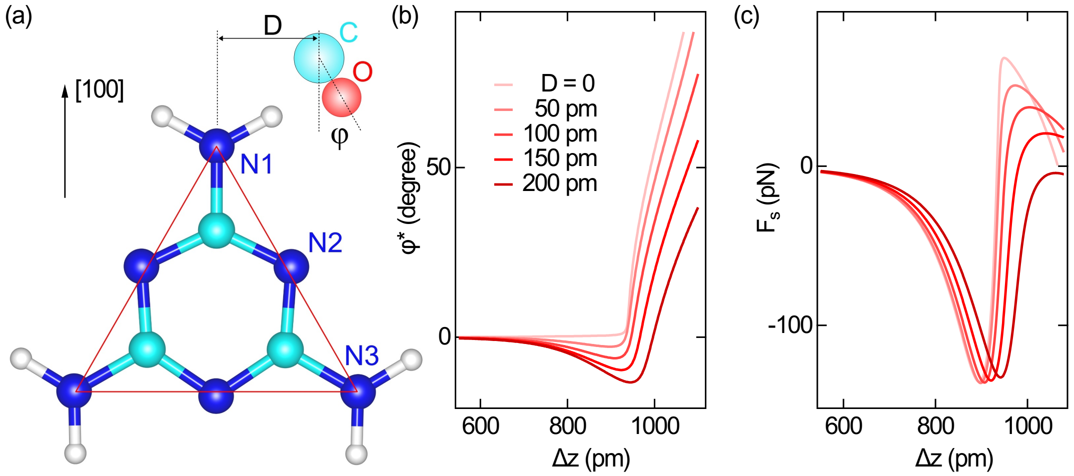

Figure S3: (a) Sketch of simplified geometry for force simulations. The MH molecule is reduced to 3 N atoms (N1, N2, N3) on one side of the equilateral triangle representing the molecule. The C–O axis is tilted by the angle φ with respect to the surface normal [100]. Distance D is defined as the separation between the C atom of the CO molecule and the symmetry axis of MH parallel to [100]. (b) Simulated angle φ^* (eq S3) of the C–O axis subtending the surface normal. Negative (positive) angle describes a bending of CO toward (away) from MH. (c) Simulated force between the O atom of CO and the three closest N atoms of MH as a function of the CO excursion Δz ($\Delta z = 0$ is defined as the distance of 2 nm between the C atom center and basis of the triangle at $D = 0$).

For each tip displacement Δz, the angle φ^* that satisfies

$$\left.\frac{\partial V}{\partial \varphi}\right|_{\varphi=\varphi^*} = 0 \tag{S3}$$

with

$$V = V_{\text{rot}} + \sum_{i=1}^{3} V_{\text{LJ},i} \tag{S4}$$

was determined for different lateral distances D (Figure S3b) by using $\varepsilon = 0.1\,\text{eV}$, $r_{\text{eq}} = 350\,\text{pm}$, $k = 0.85\,\text{N/m}$, $\ell = 113\,\text{pm}$.[6] For $\varphi^* < 0$ ($\varphi^* > 0$) the CO molecule bends toward (away from) MH. The monotonous decrease of φ^* for $D > 0$, *i. e.*, the gradual bending of CO toward MH, is due to an energy gain resulting from the attractive part of the Lennard-Jones

potential for decreasing r_i, which compensates the energy cost for bending. The variation of $\varphi^*(\Delta z)$ then traverses a minimum and intersects $\varphi^* \equiv 0$. This behavior is the result of an energy gain due to the repulsive part of the Lennard-Jones potential and the bending of CO toward the minimum of the harmonic potential. The steep monotonous increase of $\varphi^* > 0$ reflects the bending of CO away from MH. The required energy results from the energy gain upon increasing r_i in the repulsive part of the Lennard-Jones potential.

The variation of φ^* explains the evolution of the short-range force, which results from

$$F_{\mathrm{s}} = -\frac{\partial V^*}{\partial \Delta z} \tag{S5}$$

with $V^* = V(\varphi^*)$. Figure S3c shows that the experimentally observed behavior is reproduced, that is, an F_{s} attractive minimum precedes a repulsive maximum with increasing tip excursion Δz. The attractive region of F_{s} marks the tip–MH distances where the CO molecule is first tilted toward and then away from MH, while the repulsive region describes the tip–MH separations where the C–O axis adopts increasingly positive φ^*. With increasing lateral distance D the repulsion strength is attenuated in the experiments as well as in the simulations. According to the model, this observation is assigned to a weakening of the repulsive part of the Lennard-Jones potential owing to increased distances r_i.

Tip changes upon metalation

After metalation of $\mathrm{MH^T}$-α, *i. e.*, after transferring a single Cu atom from the tip to the tautomer, the qualitative comparison of STM image sharpness before and after the reaction hinted at changes of the atomic arrangement at the tip apex. A quantitative analysis is presented here by exploring I-versus-Δz traces acquired during a representative Cu tip approach to $\mathrm{MH^T}$-α (top data set in Figure S4) and, subsequently, during retraction from $\mathrm{MH^T}$-α-Cu (bottom data set in Figure S4). These data were acquired with a different Cu tip than the data depicted in Figure 6. The behavior upon approach and retraction, however, is

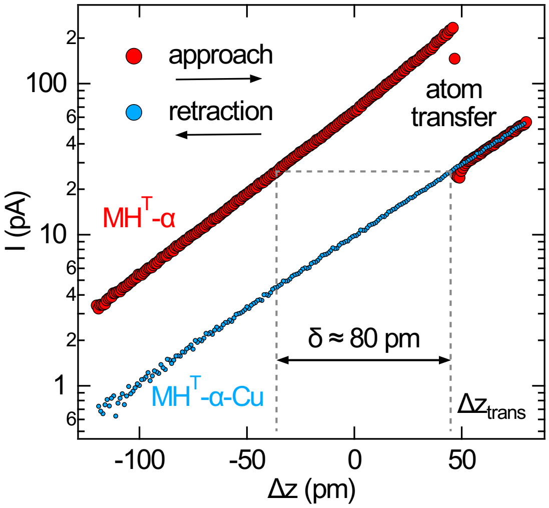

Figure S4: Current-versus-displacement traces acquired with a Cu tip during approach (top) to MH^{T}-α and retraction (bottom) from MH^{T}-α-Cu. The horizontal line links the current in the retraction data at the displacement where the Cu atom transfer occurred during tip approach ($\Delta z_{\mathrm{trans}}$) with the same current in the approach trace, while the vertical lines indicate the associated difference δ in tip displacements. Prior to data acquisition the feedback loop had been deactivated at 100 mV, 50 pm and the sample voltage was then reduced to 10 mV.

the same. The indicated Cu atom transfer ($\Delta z^{\mathrm{trans}} = 46\,\mathrm{pm}$) is accompanied by a reduction of the current across the MH^{T}-α junction by essentially one order of magnitude (from 234 pA to 24 pA). At the time resolution of the experiments the current drop appears to be abrupt. For $\Delta z > \Delta z^{\mathrm{trans}}$ the current follows the bottom trace, which belongs to the MH^{T}-α-Cu junction. The vertical offset of the two current traces is due to a reduction of the tip length by $\delta \approx 80\,\mathrm{pm}$. For this estimation, the tip displacement $\Delta z^{\mathrm{trans}}$ of the retraction trace is compared with the tip displacement of the approach data with the same current (dashed lines in Figure S4).

Reproducibility of the single-molecule metalation

The Cu metalation of MH^{T}-α presented in the article was performed by four tips that had independently been prepared by field emission and repeated indentation into the substrate surface. The successful and reproducible metalation reaction can bee seen from the evolution

of F_s and E_s shown in Figure S5. These data served as the basis for calculating the mean value and standard deviation of F_s^* and E_s^*. In detail, the extracted forces and energies at metalation read $F_s^* = -620\,\mathrm{pN}$, $E_s^* = -350\,\mathrm{meV}$ (Figure S5a), $F_s^* = -614\,\mathrm{pN}$, $E_s^* = -282\,\mathrm{meV}$ (Figure S5b), $F_s^* = -410\,\mathrm{pN}$, $E_s^* = -246\,\mathrm{meV}$ (Figure S5c), $F_s^* = -630\,\mathrm{pN}$, $E_s^* = -295\,\mathrm{meV}$ (Figure S5d).

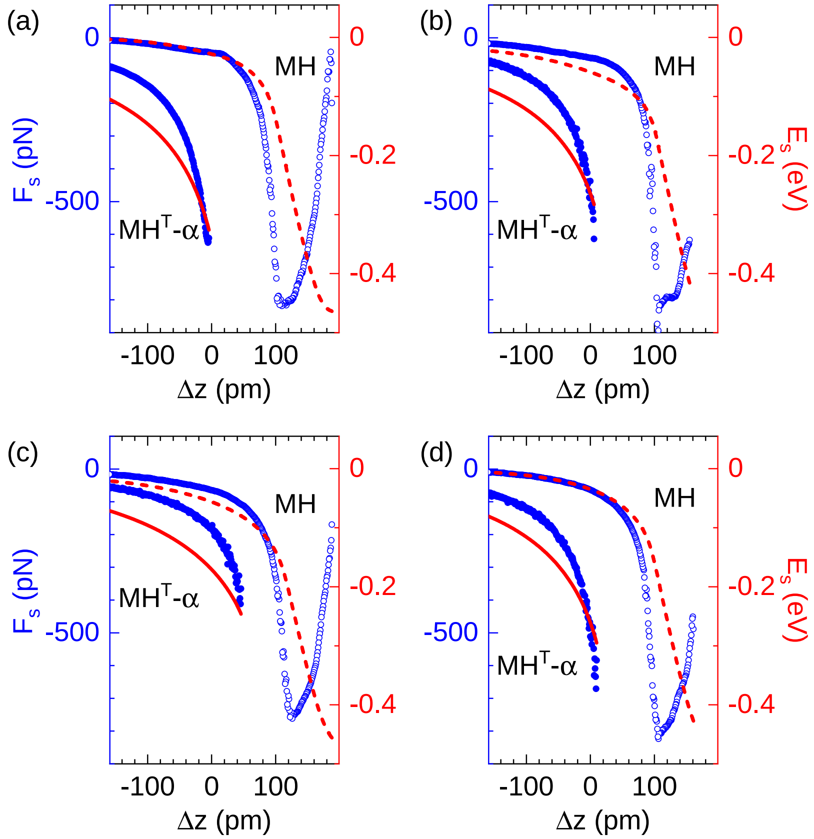

Figure S5: (a)–(d) Short-range force F_s (dots, circles) and associated energy E_s (solid and dashed lines) as a function of the Cu tip displacement Δz acquired atop $\mathrm{MH^T}$-α (left) and MH (right) acquired with four different tips. Feedback loop parameters defining $\Delta z = 0$: 100 mV, 50 pA.

In addition, Figure S6 demonstrates the reproducibility of the single-molecule metalation by a sequence of three constant-height I maps that show two $\mathrm{MH^T}$-α molecules prior to (Figure S6a) and after metalation of the top (Figure S6b) and the bottom (Figure S6c) $\mathrm{MH^T}$-α. The mutual distances of molecules in the imaged assemby as well as their relative orientation remained invariant.

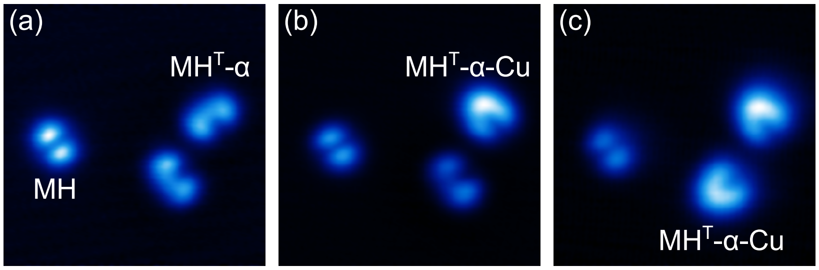

Figure S6: Sequence of constant-height I maps (3.6 nm × 3.6 nm) of MH, MH^{T}-α recorded with a Cu tip prior to (a), after the metalation of the top (b), and after the metalation of the bottom (c) MH^{T}-α. The color scale ranges from 0 (dark) to (a) 21 pA, (b) 36 pA, (c) 36 pA (bright). The feedback loop had been deactivated above Cu(100) at 100 mV and 50 pA followed by a tip retraction of 75 pm for mapping I at 10 mV.

Simulated tautomer and tautomer-Cu compounds

In addition to the molecular structures presented in Figure 1 and Figure 7, the tautomer MH^{T}-γ (Figure S7a) and the tautomer-Cu products MH^{T}-β-Cu* (Figure S7b), MH^{T}-γ-Cu* (Figure S7c) were tested in the simulations. The simulations find that demetalation of MH^{T}-β-Cu* and MH^{T}-γ-Cu* can occur with the proximity of a flat Cu tip, which, however, is at odds with experimental observations.

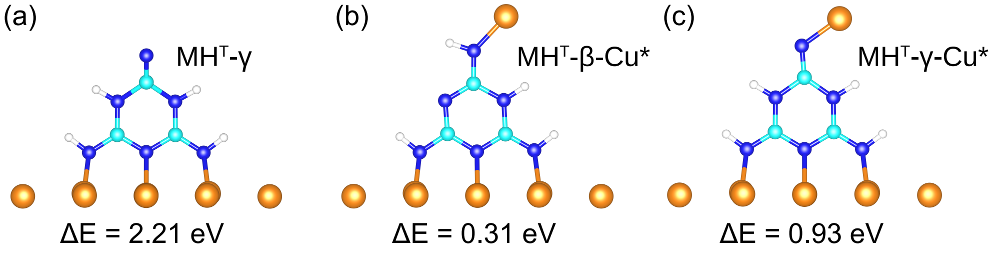

Figure S7: Additional (a) tautomer and (b),(c) tautomer-Cu compounds with indicated energy differences ΔE with respect to the ground states MH (Figure 1) and MH-Cu (Figure 7e), respectively.

Calculated constant-height electron transmission maps

Figure S8 shows the calculated transmission maps of MH and its tautomers (Figures S8a–d) as well as of MH-Cu and the tautomer-Cu compounds (Figures S8e–i). The data presented

in Figures S8a–c are consistent with previous simulations of STM images of MH and its tautomers on Cu(100).[8]

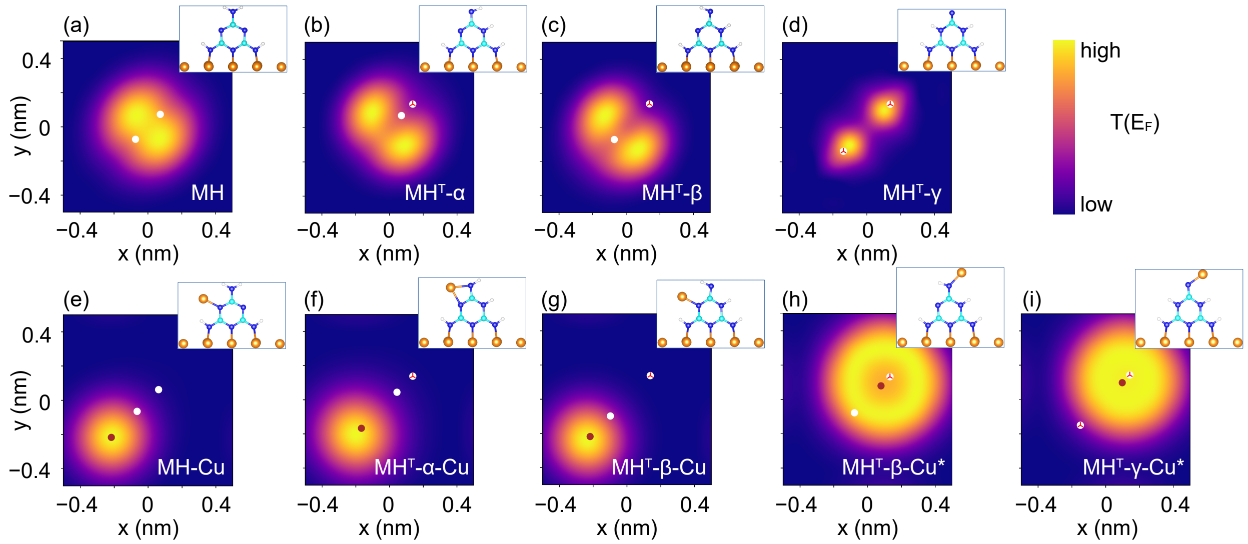

Figure S8: Maps of the elastic transmission $T(E_\mathrm{F})$ of a model junction comprising an idealized Cu tip together with MH and its tautomers (a)–(d) as well as of MH-Cu and the tautomer-Cu compounds (e)–(i) on Cu(100). The transmission is evaluated at the Fermi energy E_F and at the tip excursion $\Delta z = -300\,\mathrm{pm}$ in the far tunneling range (Figures 7a,b). White and brown dots indicate the positions of H and Cu atoms, respectively. The tautomerized H atom is additionally marked with a red symbol. The relaxed molecule structures are added as insets to all transmission maps.

In particular, the transmission patterns of MH$^\mathrm{T}$-α (Figure S8b) and MH$^\mathrm{T}$-β (Figure S8c) break the C_{2v} symmetry observed for MH (Figure S8a). The transmission maps of MH$^\mathrm{T}$-α and MH$^\mathrm{T}$-β are very similar to constant-height STM data presented in the article. The spatial variation of $T(E_\mathrm{F})$ of MH$^\mathrm{T}$-γ (Figure S8d), however, clearly deviates from the experimental results. The strong signal due to the π-orbitals that are oriented perpendicular to the molecular backbone (Figures S8a–c) is suppressed for the benefit of strong transmission close to the tautomerized H atom in this isomer.

All maps of $T(E_\mathrm{F})$ calculated for the MH-Cu and tautomer-Cu compounds (Figures S8e–i) are dominated by the electron transmission due to the s-orbital of the Cu atom, which extends deep into the vacuum and exhibits ample overlap with the Cu s-orbital of the tip.

References

1. Ternes, M.; González, C.; Lutz, C. P.; Hapala, P.; Giessibl, F. J.; Jelínek, P.; Heinrich, A. J. Interplay of Conductance, Force, and Structural Change in Metallic Point Contacts. *Phys. Rev. Lett.* **2011**, *106*, 016802.

2. Ladenthin, J. N.; Frederiksen, T.; Persson, M.; Sharp, J. C.; Gawinkowski, S.; Waluk, J.; Kumagai, T. Force-Induced Tautomerization in a Single Molecule. *Nat. Chem.* **2016**, *8*, 935 – 940.

3. Liebig, A.; Giessibl, F. J. In-Situ Characterization of O-Terminated Cu Tips for High-Resolution Atomic Force Microscopy. *Appl. Phys. Lett.* **2019**, *114*, 143103.

4. Giessibl, F. J. A Direct Method to Calculate Tip–Sample Forces from Frequency Shifts in Frequency-Modulation Atomic Force Microscopy. *Appl. Phys. Lett.* **2001**, *78*, 123–125.

5. Sader, J. E.; Jarvis, S. P. Accurate Formulas for Interaction Force and Energy in Frequency Modulation Force Spectroscopy. *Appl. Phys. Lett.* **2004**, *84*, 1801–1803.

6. Néel, N.; Kröger, J. Atomic Force Extrema Induced by the Bending of a CO-Functionalized Probe. *Nano Lett.* **2021**, *21*, 2318–2323.

7. Welker, J.; Giessibl, F. J. Revealing the Angular Symmetry of Chemical Bonds by Atomic Force Microscopy. *Science* **2012**, *336*, 444–449.

8. Pan, S.; Fu, Q.; Huang, T.; Zhao, A.; Wang, B.; Luo, Y.; Yang, J.; Hou, J. Design and Control of Electron Transport Properties of Single Molecules. *Proceedings of the National Academy of Sciences* **2009**, *106*, 15259–15263.
